# Deciphering the maturation of tertiary lymphoid structures in cancer and inflammatory diseases of the digestive tract using imaging mass cytometry

**DOI:** 10.3389/fimmu.2023.1147480

**Published:** 2023-04-18

**Authors:** Marion Le Rochais, Patrice Hémon, Danivanh Ben-guigui, Soizic Garaud, Christelle Le Dantec, Jacques-Olivier Pers, Divi Cornec, Arnaud Uguen

**Affiliations:** ^1^ Lymphocytes B, Autoimmunité et Immunothérapies (LBAI), Unité Mixte de Recherche (UMR)51227, Univ Brest, Inserm, Brest, France; ^2^ Centre Hospitalo-Universitaire (CHU) de Brest, Brest, France

**Keywords:** tertiary lymphoid structures, imaging mass cytometry, Hyperion, cancer, inflammation

## Abstract

Persistent inflammation can promote the development of tertiary lymphoid structures (TLS) within tissues resembling secondary lymphoid organs (SLO) such as lymph nodes (LN). The composition of TLS across different organs and diseases could be of pathophysiological and medical interest. In this work, we compared TLS to SLO in cancers of the digestive tract and in inflammatory bowel diseases. Colorectal and gastric tissues with different inflammatory diseases and cancers from the department of pathology of CHU Brest were analyzed based on 39 markers using imaging mass cytometry (IMC). Unsupervised and supervised clustering analyses of IMC images were used to compare SLO and TLS. Unsupervised analyses tended to group TLS per patient but not per disease. Supervised analyses of IMC images revealed that LN had a more organized structure than TLS and non-encapsulated SLO Peyer’s patches. TLS followed a maturation spectrum with close correlations between germinal center (GC) markers’ evolution. The correlations between organizational and functional markers made relevant the previously proposed TLS division into three stages: lymphoid-aggregates (LA) (CD20+CD21-CD23-) had neither organization nor GC functionality, non-GC TLS (CD20+CD21+CD23-) were organized but lacked GC’s functionality and GC-like TLS (CD20+CD21+CD23+) had GC’s organization and functionality. This architectural and functional maturation grading of TLS pointed to differences across diseases. TLS architectural and functional maturation grading is accessible with few markers allowing future diagnostic, prognostic, and predictive studies on the value of TLS grading, quantification and location within pathological tissues in cancers and inflammatory diseases.

## 1 Introduction

Inflammation is the immune system’s response to various diseases, such as cancer, autoimmune diseases, and infections occurring within secondary lymphoid organs (SLO), particularly in draining lymph nodes (LN). In case of persistent inflammation, the migration and positioning of immune cells follow the organogenesis of SLO within organs, to give rise to tertiary lymphoid structures (TLS) detectable as nodular lymphoid-aggregates (LA) at the microscopic level (1–3). TLS can develop in infectious, autoimmune, and inflammatory diseases, as well as in solid cancers with a potential significance in terms of prognosis and response to treatments (4, 5). Studies on several diseases causing chronic inflammation proposed staging classifications of TLS depending notably on the density of CD20+ B cells and the formation of a follicular structure in TLS (6–8). But authors have used different structural, cell differentiation, and functional markers to describe various degrees of TLS, often with limited and heterogeneous panels of markers. This methodological heterogeneity in TLS staging is a limitation for the comparisons of data between studies. A clearer and more reproducible definition of what is a TLS and how mature it is regarding its cell composition, organization, and functionality is needed to better investigate the diagnostic, prognostic, and predictive values of TLS analysis.

TLS are particularly frequent in various digestive tract diseases. As bacterial and viral infections have been reported in the initiation of TLS formation at the site of infection, TLS are notably observed in the stomach in case of Helicobacter pylori inducing chronic gastritis (9). TLS are also observed in chronic inflammation caused by autoimmune diseases (such as Biermer’s disease – autoimmune gastritis) or inflammatory diseases (Crohn’s disease, Chronic diverticulitis, and Ulcerative colitis) (10, 11). Their role in inflammation and autoimmunity is difficult to establish: in murine models, TLS could have a dual function, with opposite pathogenic effects (12). Indeed, TLS could play a role in pathogen clearance beneficial for the patient, as well as being sites of autoimmune response amplifications and tissue damage aggravations leading to a deleterious disease evolution (13).

In the cancer field, TLS could also play a dichotomous role in tumor invasion control and metastasis. TLS are privileged sites for antigen presentation to B cells at the tumor site, leading to the increase of the local production of antitumor immunoglobulins G, helping in tumor control and improving patient’s prognosis (13). Contrastingly, TLS can recruit immunosuppressive cells and become immunological micro-niches promoting the generation of progenitor cancer cells leading to deleterious outcomes (14). Thus, the prognostic significance of TLS remains unclear. Interestingly, in some colorectal and gastric cancers with an impaired DNA mismatch repair (causing a “microsatellite instability” MSI status), the resulting increased number of neoantigens related to numerous mutations gives rise to highly immunogenic tumors, notably rich in TLS. MSI tumors are of different prognosis in comparison with tumors lacking this deficiency (called “stable microsatellites” (MSS)). Contradictory data are reported about MSI tumors with a better prognosis at an early stage but a poorer prognosis in advanced/metastatic MSI tumors. Due to their high immunogenicity, the growth of MSI tumors is conditioned by a high level of immunomodulation, so they are good responders to targeted immune checkpoints inhibitors treatments. Furthermore, regardless of patient characteristics, MSI status and TLS presence generally correlate with improved survival in CRC patients (7, 15, 16).

The frequency of TLS in various gastric and colonic diseases makes stomach and colon predilection sites for a better characterization of TLS. However, the small size and unpredictable location of TLS within the tissues, detectable only on microscopic examination of tissues, are limitations for their detailed analysis. Thus, highly multiplexed methods are required to extract in-depth data from minimal tissue areas selected at the histopathological microscopic level. The recent imaging mass cytometry (IMC) technique allows to co-analyze nearly 40 markers concomitantly in the same tissue section with increasing applications especially in cancer research (17, 18). IMC is particularly appropriate to analyze in-depth the phenotypes and spatial organization of cells in small tissular structures such as TLS.

The objectives of the present work were to decipher and compare the compositions and organizations of TLS observed in different inflammatory and cancerous, gastric and colorectal diseases using IMC.

## 2 Materials and methods

### 2.1 Cases and sample selection

Tissue samples from patients analyzed for care purpose in the department of pathology of CHU Brest were used, following our national and institutional guidelines, in compliance with the Helsinki Declaration and after approval by our institutional review board. All samples were included in the registered tissue collection part of the Brest Biological Resources Center BB-0033-00037, NF S 96-900 certificated Brest, CHRU Brest AC-2019-3642 - DC – 2008 – 214). No clinical, epidemiological or evolution data about the patients was collected during this study. Groups of patients with gastric samples were constituted as follows: (A) control patients with gastric weight-reduction surgery with lymphoid islet with healthy mucosa; (B) patients with autoimmune gastritis (Biermer disease); (C) patients with Helicobacter pylori–related gastritis; (D) patients with MSI gastric adenocarcinoma, differentiating those without nodal metastasis (D1) from those with nodal metastasis (D2); (E) patients with MSS gastric adenocarcinoma without nodal metastasis (E1) or with nodal metastasis (E2). In the same manner for colorectal tissues (1):: control patients with pT0N0 status colectomy specimens complementary to endoscopic resection of pT1 colorectal cancers, Peyer’s patches were selected distant to the resection site next to the resection margins (2); patients with Crohn’s disease (3); patients with ulcerative colitis (4); patients with chronic diverticulitis (5); patients with MSI colonic adenocarcinoma differentiating those without nodal metastasis (5A) from those with nodal metastasis (5B) (6); patients with MSS colonic adenocarcinoma without nodal metastasis (6A) or with nodal metastasis (6B). For patients with gastric or colonic adenocarcinomas, one regional LN without nodal metastasis was also selected, completed by a LN with nodal metastasis for metastatic patients (**Table 1**). The costs and workflows constraints of IMC technology prompted us to design a highly qualitative study selecting only small groups of patients with different diseases (3 to 6 patients per group). Formalin-fixed paraffin-embedded (FFPE) tissue samples and corresponding tissue slides were collected: areas containing TLS were selected on histopathological slides and IMC analyses were performed on new tissue sections.

**Table 1:** Cases and regions of interest selected for imaging mass cytometry.

Organs	Group	Condition	Cases number	ROI number
Stomach	A	No gastric disease	6	14
	B	Biermer’s autoimmune gastritis	4	10
	C	*Helicobacter pylori* –related gastritis	5	8
	D D1 D2	Microsatellite instable gastric adenocarcinoma Non metastatic (nm) Metastatic (m)	6 3 3	45 18 (11T, 7LN) 27 (14T, 5LN, 8LNm)
	E E1 E2	Microsatellite stable gastric adenocarcinoma Non metastatic (nm) Metastatic (m)	6 3 3	53 18 (12T, 6 LN) 35 (21T, 7LN, 7LNm)
	TOTAL		27	130
Colon	1	Peyer’s patches	5	11
	2	Crohn’s disease	5	19
	3	Ulcerative colitis	5	14
	4	Chronic diverticulitis	5	10
	5 5A 5B	Microsatellite-instable colonic adenocarcinoma Non metastatic (nm) Metastatic (m)	6 3 3	55 25 (18T, 7LN) 30 (12T, 8LN, 10LNm)
	6 6A 6B	Microsatellite-stable colonic adenocarcinoma Non metastatic (nm) Metastatic (m)	6 3 3	50 15 (4T, 11LN) 35 (10T, 9LN, 16LNm)
	TOTAL		32	159

ROI, region of interest selected for imaging mass cytometry analysis; T, primary tumor site; LN, non-metastatic lymph node; LNm, metastatic lymph node.

### 2.2 Selection of a 39 markers-panel for TLS description

We developed and validated an IMC panel of 39 metal-tagged antibodies, composed of structural, functional, and immune markers for the in-depth description of TLS. 7 immune markers were selected for T cell populations (CD3/CD4/CD8/Tbet/GATA3/Stat3/FoxP3), 4 immune markers for B cell and plasma cell populations (CD20/CD138/CD38/CD23/CD27), and 9 other markers for DC, macrophages, granulocytes, and NK cells. 5 markers (CD31/CD34/Podoplanin/Pankeratin/CD103) were selected as non-immune markers to enhance visualization of tissue architecture: vessels, epithelium, and fibroblasts. We also selected 13 functional markers (Bcl6/AID/HLADR/CXCR5/CD86/Ki67/Cleaved-Caspase-3/PD-1/PD-L1/CD27/IgM/IgD/IgG) to evaluate the TLS functions in terms of activation, proliferation, inhibition, checkpoint, cytokines and immunoglobulins. A nuclear intercalator dye (Iridium) was included to allow the identification and segmentation of individual cells in our analysis pipeline **(** **Table 2** **).**

**Table 2:** Panel of markers and corresponding metals-labeled antibodies for tertiary lymphoid characterization.

Marker		Metal		Supplier	Dilution	Antibody clone	Metal coupling: in-house (I) or manufacturer (M)
**CD83**		139La		Abcam	1/100	EPR23809-19	I
**CD38**		141Pr		Fluidigm	1/100	EPR4106	M
**CD21**		142Nd		Abcam	1/400	SP186	I
**CD23**		143Nd		Abcam	1/500	EPR3617	I
**DCLAMP**		144Nd		Novus	1/50	1010E1.01	I
**T-bet**		145Nd		Fluidigm	1/50	D6N8B	M
**CD34**		146Nd		Abcam	1/200	SP179	I
**CD163**		147Sm		Fluidigm	1/200	EDHu-1	M
**Pankeratin**		148Nd		Fluidigm	1/200	C11	M
**GATA-3**		149Sm		Abcam	1/300	EPR16651	I
**CD274 (PD-L1)**		150Nd		Abcam	1/75	SP142	I
**CD31**		151Eu		Fluidigm	1/100	EPR3094	M
**Ki-67**		152Sm		Abcam	1/300	MKI67/2642	I
**IgD**		153Eu		Southern Biotech	1/40	Polyclonal	I
**AID**		154Sm		Abcam	1/500	EPR23436-45	I
**FoxP3**		155Gd		Abcam	1/50	236A/E7	I
**CD86**		156Gd		RD	1/100	Polyclonal	I
**CD68**		159Tb		Fluidigm	1/400	KP1	M
**Bcl-6**		160Yb		Abcam	1/75	CAL49	I
**CD20**		161Dy		Fluidigm	1/400	H1	M
**CD8a**		162Dy		Fluidigm	1/200	D8A8Y	M
**CD138**		163Dy		Abcam	1/400	SP152	I
**MPO**		164Dy		Abcam	1/1000	EPR20257	I
**CD279 (PD-1)**		165Ho		Abcam	1/100	NAT105	I
**CD56**		166Er		Abcam	1/50	NCAM1/1496	I
**Granzyme (GzB)**		167Er		Fluidigm	1/200	EPR20129-217	M
**CXCR5**		169Er		Abcam	1/100	EPR23463-30	I
**CD3**		170Er		Fluidigm	1/150	Polyclonal	M
**CD27**		171Yb		Fluidigm	1/100	EPR8569	M
**Caspase-3 cleaved**		172Yb		Fluidigm	1/100	5A1E	M
**Podoplanin**		173Yb		Abcam	1/400	PDPN/1433	I
**HLA-DR**		174Yb		Fluidigm	1/600	YE2/36HLK	M
**CD4**		175Lu		Abcam	1/300	EPR6855	I
**IgM**		176Gd		Dako	1/100	Polyclonal	I
**Cell intercalator**		191/193Ir		Fluidigm	1/500	-	M
**CD11c**		194Pt		Abcam	1/300	ITGAX/1242	I
**CD103**		195Pt		Abcam	1/75	SP301	I
**Stat3**		196Pt		Abcam	1/800	EPR787Y	I
**IgG**		209Bi		Dako	1/150	Polyclonal	I

### 2.3 Antibodies validation using immunohistochemistry

For the antibodies not already conjugated to metals by the manufacturer, the antibodies performances and optimal antigen retrieval conditions were assessed by chromogenic immunohistochemistry (IHC). Tissue sections were deparaffinized and rehydrated with xylene and decreasing concentrations of ethanol. Sections were boiled for 20min in Tris-EDTA (10 mM/1 mM, pH 9) buffer for antigen retrieval, followed by endogenous peroxidase blockade using a 0.3% H2O2 solution for 5min and non-specific antibody binding blockade with 3% TBS BSA solution for 30min. Tissues were incubated overnight at 4°C with the primary antibody. Following washes in PBS, tissues were incubated with a secondary antibody coupled to horseradish peroxidase (Polink-1 HRP for Rabbit & Mouse - GBI Labs Kit/AffiniPure Goat Anti-Rat IgG -112-005-143, Jackson/AffiniPure Donkey Anti-Goat IgG - 705-005-003, Jackson) for 1h at room temperature. Antibody binding was detected with DAB+ chromogen (DAKO, Agilent technologies, Santa Clara, Ca, USA) and the sections were counterstained with hematoxylin (Thermo Fisher Scientific, Waltham, Massachusetts, USA), before dehydration and mounting of the slides. The specificities and intensities of the immunostaining were assessed by a pathologist.

### 2.4 Antibodies and metal conjugation

IHC-validated antibodies were conjugated to purified lanthanide metals (Fluidigm, San Francisco, CA, USA) (**Table 2**) using the MaxPar antibody labeling kit (Fluidigm) according to the manufacturers’ instructions. After conjugation, all coupled antibodies were eluted in antibody stabilizer buffer (Candor Bioscience, Wangen, Germany). The titration of the coupled antibody in IMC was done on the positive tissue to determine the optimal concentration. Three different concentrations, were tested (0.5X, 1X, and 2X where 1X was defined according to the antibody’s concentration in IHC). The best concentration was then chosen to have the highest staining intensity with the least noise background.

### 2.5 Tissue labeling before imaging mass cytometry acquisition

For downstream analyses, all samples were cut at 3 μm and placed on Superfrost^®^ Plus slides (Thermo Scientific, Saint-Herblain, France). All slides were stained with alcian blue to record the microscopic morphology of the tissue. Slides were scanned at 20X magnification on the 3DHistech Panoramic Midi slide scanner (3DHISTECH, Budapest, Hungary), and visualized using CaseViewer software (v.2.4/32bits, 3DHistech) for the selection of ROIs (**Supplementary Figure 1**). With successive xylene baths, coverslips were removed and the mounting medium was washed. The same IHC-protocol was followed except for the peroxidase blockade step, before the incubation of slides with the 39 metal-conjugated antibodies panel and the cell intercalator.

### 2.6 Imaging mass cytometry acquisition

Before the acquisition, the Hyperion mass cytometry system (Fluidigm) was autotuned using a 3-element tuning slide (Fluidigm) according to the tuning protocol provided by Fluidigm. ROIs with sizes ranging from 300 x 300 µm to 1,200 x 1,200 µm were ablated and acquired at 200 Hz. For each sample, one or more ROI were defined for the acquisition on Hyperion, depending on the number of TLS on slides.

### 2.7 Image analysis pipeline based on QuPath

Generated data were visualized as MCD files using the Fluidigm MCDTM viewer. To better separate antibodies signals and noises, each marker was separately visualized and a minimum signal threshold was set in the Fluidigm MCDTM viewer. For each recorded ROI, a stack of 16-bit single-channel OME-TIFF files was exported from MCD binary files using MCDTM Viewer 1.0 (Fluidigm). OME-TIFF files of each marker were then stacked in one TIFF file with ImageJ (v.1.8.0_172). The TIFF files were opened with QuPath (v.0.3.2), setting the image type on fluorescence, for running the segmentation with the best parameters for each ROI, based on the nuclei-staining Iridium channel and peri-nuclear cell expansion (19). Measurements (mean of the marker signal in each cell) were then exported as a CSV table for analysis.

### 2.8 Unsupervised and supervised clustering analyses

The unsupervised clustering was made with Cytofkit, a Bioconductor package. The cellular subsets detection was realized by the clustering algorithm PhenoGraph (nearest neighbors k=120). Cytofkit is implemented in R, licensed under the Artistic license 2.0, and freely available from the Bioconductor website, https://bioconductor.org/packages/cytofkit/. The supervised clustering was based on the 15 following markers: CD103, CD138, CD163, CD20, CD27, CD3, CD34, CD4, CD56, CD68, CD8, DCLAMP, MPO, PanKeratin, Podoplanin. No transformation method was used, and a t-sne dimension reduction was applied.

### 2.9 Pathologic assessment of IMC images

A semi-quantitative supervised analysis was performed with the visual grading of IMC images through 15 markers representative of the germinal center (GC) (DCLAMP/CD3/CD8/CD20/CD21/CD23/CD4/

IgD/CXCR5/CD38/Podoplanin/AID/Bcl6/CD68/CD138). The scoring was performed by a pathology-trained scientist blinded to the diagnostic and experimental data. Each marker was analyzed for each ROI (TLS+LN) and scored from 0 to 4 according to the organization: 0 being the absence of staining and 4 the highest grade of organization, with an aspect very close to a functional GC as expected in LN (**Table 3** and **Supplementary Figure 2**). Correlations scores between markers were determined. If the p-value was significant (p<0.05), correlations scores higher than 0.5 were considered as low positive and correlations scores higher than 0.7 were considered as high positive. Statistics and heat maps were generated in R (v4.0.5) with the ggplot2 package.

**Table 3:** Criteria used for the pathological assessment of germinal center markers on imaging mass cytometry images.

Markers	Possible scores
**Structural markers** DC LAMP, CD3, CD8, CD20, CD21, CD23, CD4, IgD **Functional markers** CXCR5, CD38, Podoplanin, AID	• 0: absence of staining • 1: a few scattered stained cells • 2: non-nodular layer • 3: crown/nodule organization • 4: dense crown organized around the nodule
**Nodular interactions markers** CD68, CD138, Bcl6	• 0: absence of staining • 1: staining outside the nodule • 2: staining inside the nodule • 3: staining outside and inside the nodule

In addition, TLS were classed according to the 3 stages classification in terms of LA (CD20+CD21-CD23- TLS), Non-GC TLS (CD20+CD21+CD23-) and GC-like TLS (CD20+CD21+CD23+). Distributions between groups were compared using the Kruskal-Wallis non-parametric test performed on Medcalc software.

## 3 Results

### 3.1 Cases and ROIs selections

A total of 130 ROIs were selected from 27 gastric samples and 159 additional ROIs were selected from 32 colonic samples (**Table 1**).

### 3.2 Unsupervised single-cell analysis reveals a patient-dependant TLS clustering

An unsupervised analysis was run to determine if there were similar cellular clusters between the TLS of the same organ, the same disease, or a group of affection (infectious, inflammatory, cancer). The RPhenograph analysis was applied to all ROI (excepting those concerning LN, 90 gastric ROI and 98 colonic ROI) to identify meta-clusters associated with distinct subpopulations and the dendrogram enabled to group TLS expressing same cellular clusters.

**Figure 1:**
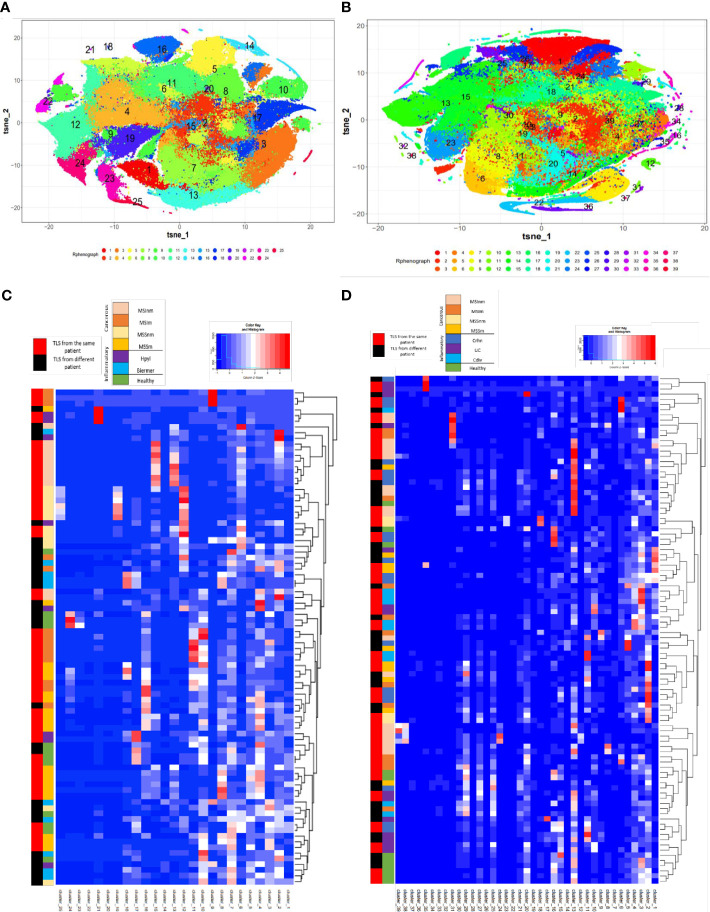
T-sne plots and the corresponding heatmaps of the cellular clusters obtained with Rphenograph composing TLS in gastric and colonic samples. **(A)** T-Sne plot showing the cellular composition of each TLS in gastric samples (excluding LN) composed of 25 clusters. **(B)** T-Sne plot showing the cellular composition of each TLS (excluding LN) composed of 39 clusters in colonic samples. **(C)** Heatmap about gastric data shows median marker expression of clusters detected by RPhenoGraph in TLS coming from the different conditions 1) healthy, 2) inflammatory: Hpyl = patient with infection by Helicobacter pylori, Bierm = patient with Biermer’s disease, and 3) cancerous: patients with adenocarcinoma of MSI- or MSS- status, with a non-metastatic (-nm) or metastatic stage (-m). **(D)** Heatmap about colonic data shows median marker expression of clusters detected by RPhenoGraph in TLS coming from the different conditions 1) healthy, 2) inflammatory: Crhn = patient with Crohn’s disease, UC = patient with ulcerative colitis, and Cdiv = patient with chronic diverticulitis, and 3) cancerous: patients with adenocarcinoma of MSI- or MSS- status, with a non-metastatic (-nm) or metastatic stage (-m). For **(C, D)** heatmaps, row labels represent the cluster IDs and column labels show the TLS name. The multicolor line on the left represents the grouping of TLS according to their conditions where each color is specific for a condition. The red and black line on the left represents in red the TLS that are closely grouped within the dendrogram and found in the same patient whereas in black are TLS from different patients.

This identified 25 distinct meta-clusters for the stomach (**Figure 1A**) and 39 for the colon (**Figure 1B**) enabling the visualization of a heatmap plot with its dendrogram where TLS from the same condition were not grouped. TLS cellular composition appeared to be more patient-dependent, rather than disease-dependent because no cluster was found more specifically in one disease or another. For gastric samples, a majority of TLS from the same sample and patient were grouped within the dendrogram (55/90 (61%) TLS, **Figure 1C**). We found the same for colonic samples with a majority of TLS from the same patient grouped within the dendrogram (60/98 (61%) TLS, **Figure 1D**). Given this analysis, TLS cellular compositions does not seem to be dependent on the disease or the organ but could be more dependent on the particular features of each patient’s immune system and proper pathology.

### 3.3 Semi-quantitative analysis based on 15 GC markers highlights a structural organization more important in LN than in TLS

**Figure 2:**
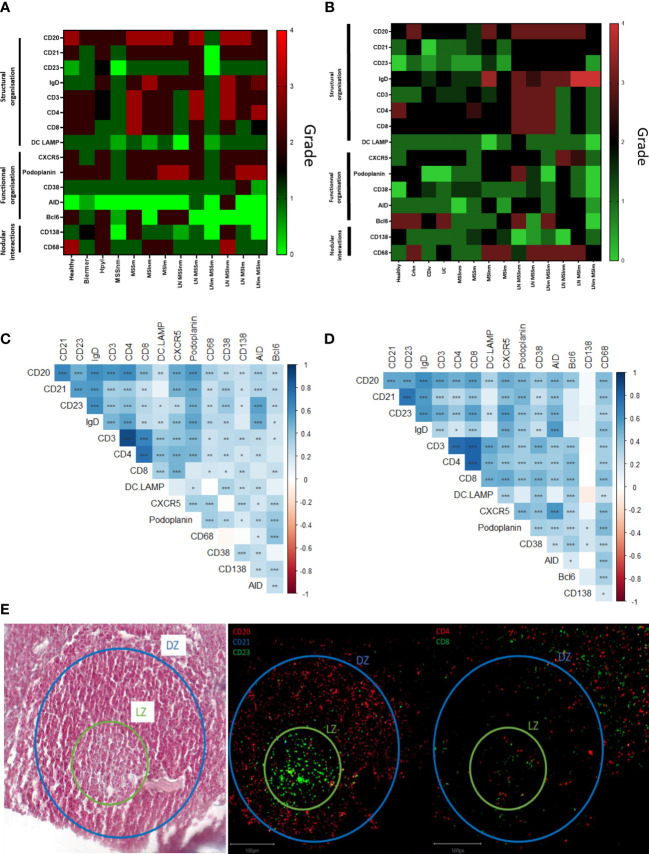
Semi-quantitative supervised analysis based on the scoring of 15 markers characterizing germinal centers and correlations between these markers in the TLS from gastric and colonic samples. **(A)** Heatmap representation of the average score observed for each marker for the different conditions observed in the stomach: 1) healthy, 2) inflammatory: Hpyl = patient with infection by Helicobacter pylori, Bierm = patient with Biermer’s disease, and 3) cancerous: patients with adenocarcinoma of MSI- or MSS- status, with a non-metastatic (-nm) or metastatic stage (-m). **(B)** Heatmap representation of the average score observed for each marker for the different conditions observed in the colon: 1) healthy, 2) inflammatory: Crhn = patient with Crohn’s disease, UC = patient with ulcerative colitis, and Cdiv = patient with chronic diverticulitis, and 3) cancerous: patients with adenocarcinoma of MSI- or MSS- status, with a non-metastatic (-nm) or metastatic stage (-m). The follicles from LN without nodal metastasis (LN) and with nodal metastasis (LNm) are also included in the analysis revealing a more mature structural organization in LN than in TLS. **(C)** Heatmap representation of the correlation between the markers in gastric TLS. **(D)** Heatmap representation of the correlation between the markers in colonic TLS. Correlated and uncorrelated markers are shown in dark blue to red respectively. Closer are the markers on the figure, higher are their correlation. Significative p-values are represented with stars: * = p<0.05, ** = p<0.01, *** = p< 0.001. **(E)** IMC images of the light (LZ) and dark zone (DZ) in a GC of a TLS from a Crohn’s disease sample with the corresponding HE image on the left.

To better understand the development of the GC in TLS and make a comparison with the classical GC found in LN, we made a pathologic assessment of IMC images. We decided to grade 15 chosen markers characterizing GC organization and functions: CD20/CD21/CD23/IgD/CD3/CD4/CD8/DCLAMP/CXCR5/Podoplanin/CD38/AID/Bcl6/CD138/CD68. In gastric samples, for the 8 structural markers, a difference could be observed between the lymphoid follicles from the LN and TLS from cancerous tissues. This was also the case between TLS from the inflammatory conditions and controls. Controls had higher scores (majority of 3 to 4) for the structural markers than the others (**Figure 2A**). The same trend was observed in colonic samples, with a difference between the LN and TLS from the different conditions (**Figure 2B**), with a higher score for LN independently from their status (metastatic or not) than in TLS, highlighting that LN are structurally better organized than TLS. Of note, SLO Peyer’s patches were closer to TLS than to other SLO (LN).

### 3.4 Semi-quantitative analysis on non-grouped TLS scoring emphasizes two different areas in TLS

To understand markers associated with the TLS organization, we assessed the correlation between the 15 markers in individual TLS (**Figures 2C, D**).

In the stomach (**Figure 2C**), the correlated markers were the T-cells markers such as CD3/CD4 (0.898) and CD4/CD8 (0.746), structural markers of the GC (CD20/CD21/CD23/IgD) and functional ones (podoplanin/AID/CXCR5) (**Figure 2C** and **Supplementary Figure 3A**). The dendrogram grouped the positive correlations occurring between the marker of T cells (CD3, CD4, CD8) and DCLAMP, markers found in the T-zone of the TLS. We also noted a correlation with the functional marker CXCR5, suggesting the presence of Tfh (T follicular helper) and Tfr (T follicular regulator). In another branch of the dendrogram, markers of B cells and heart of the GC (B-zone): CD20, CD21, CD23, IgD and podoplanin were grouped and presented a low positive correlation (**Figure 2C**).

In the colon, the same trends were observed. The dendrogram grouped positive correlations occurring between organizational markers of the T cell zone (CD3/CD4/CD8/CD38/DCLAMP) and the functional marker Bcl6. We had another group of markers correlated representing the B-zone with organizational markers (IgD/CD21/CD20/CD23/CD68), or functional markers such as AID and CXCR5. The marker CD138 did not appear to correlate with either of these two groups (T-zone and B-zone) but still appeared to correlate with CD38 and podoplanin (**Figure 2D** and **Supplementary Figure 3B**).

In this manner, for both organs, the same correlated structural markers were observed within two different groups which can be differentiated in B- and T-zone in the TLS composition. The B-zone corresponds to the light zone (LZ) and the dark zone (DZ) of the GC. The LZ consisted of the FDC network with or without the active GC (CD23+) depending on the maturation stage of the TLS. The dark zone corresponding to the non-proliferative B cells (CD20+ CD23-) which are surrounded by T cells (CD4+ and CD8+ ones) (**Figure 2E**). The T-zone contained mature DCLAMP+ dendritic cells (DC), adjacent to a B-zone which included a typical GC comprising B cells embedded within a network of follicular DC.

### 3.5 Functional description of E-TLS, PFL-TLS and SFL-TLS and quantifications across gastric and colonic diseases

**Figure 3:**
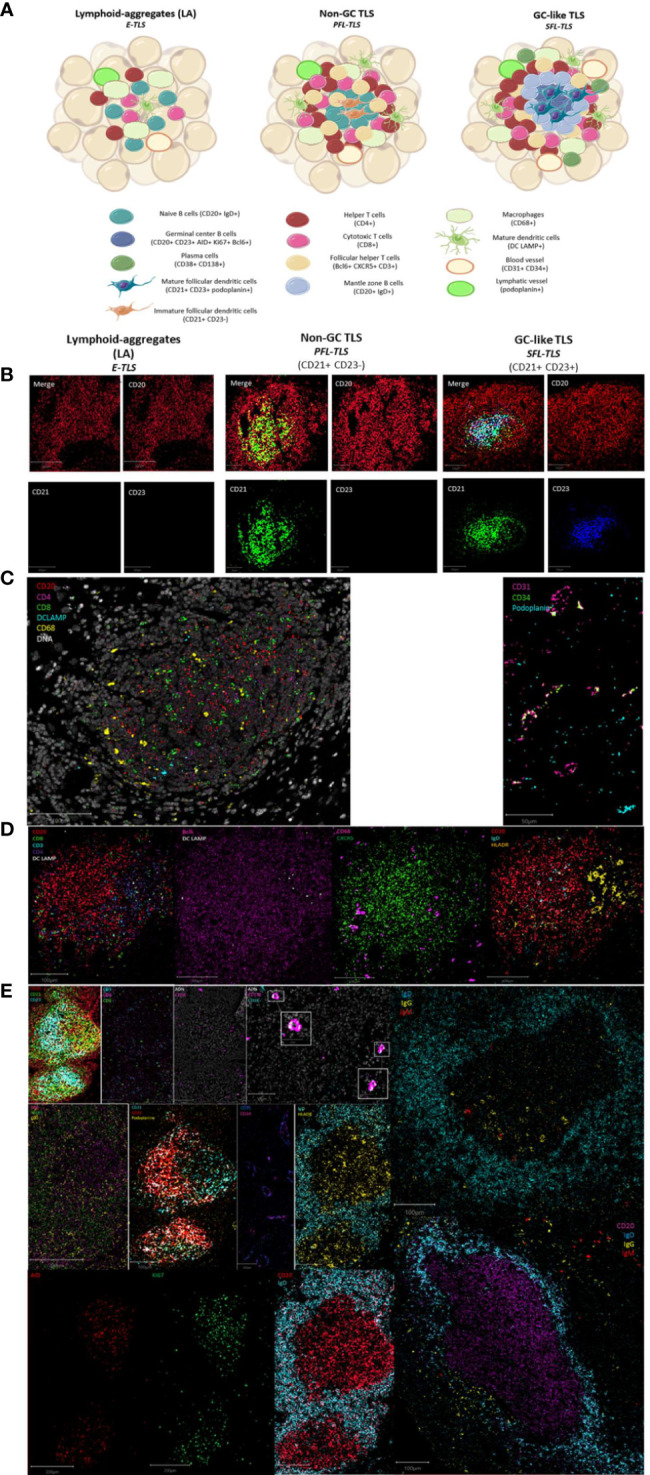
Maturation stages of tertiary lymphoid structures. **(A)** The different stages of tertiary lymphoid structures. Lymphoid-aggregates (LA) constituted of T and B cells, with some macrophages and mature dendritic cells, corresponding to early-TLS stage in Silina’s work. Non-germinal center (GC) TLS, corresponding to primary follicle-like TLS, is formed with distinct organized T and B cells zones including follicular dendritic cells in the B-zone. Germinal center (GC)-like TLS, corresponding to secondary follicle-like TLS, is a functional germinal center composed of proliferating mature germinal center B lymphocytes (AID+Bcl6+Ki67+) and follicular dendritic cells (podoplanin+) surrounded by mantle zone B cells (IgD+) and bordered by follicular helper T cells (Bcl6+CXCR5+CD3+). In addition, TLS are formed of CD4+ and CD8+ T cells, plasma cells, mature dendritic cells, and macrophages enriched in the T-zone. **(B)** Representative images of the IMC markers CD20, CD21 and CD23 characteristics of the three different TLS-stages. Images from the three TLS come from the same Crohn’s patient. **(C)** Representative image of IMC markers found in immune aggregate of a patient with Biermer disease. **(D)** Representative images of IMC markers found in non-GC TLS of a Crohn’s patient. **(E)** Representative images of IMC markers found in GC-like TLS found in one colonic MSI adenocarcinoma and two gastric MSS samples. White boxes show the plasma cells (CD38+CD138+) appearing as a colocalization of a cyan and a pink signal.

Beyond a globally less-organized structure of TLS compared to LN and correlations in terms of markers of the light and the dark zones within TLS, the morphological analysis of IMC images in our study revealed heterogeneous organizations ranging from poorly-organized LA to well-organized structures forming follicles or even functional GC in different organs and diseases. Classifying the TLS into three stages lymphoid-aggregates (LA), Non-GC TLS and germinal center GC-like TLS, we observed the following correlations:

Dense LA, corresponding to the E-TLS stage from Silina et al. (8), lacking CD21+ FDCs and CD23+ GC which were disorganized LA with T cells (CD3+CD4+ or CD3+CD8+) and B cells (CD20+), mature DC (DCLAMP+) and some macrophages (CD68+) surrounding lymphatic (podoplanin+) and blood vessel (CD31+CD34+) (**Figures 3A–C**).

Non-GC TLS defined as LA with immature CD21+ FDCs but no CD23+ GC reaction, corresponding to the PFL-TLS stage (8). They had a nodular organization with a B cell nodule composed of naïve B cells (IgD+CD20+) and follicular helper T cells (Bcl6+CXCR5+CD3+). The B cell zone was surrounded by a T cell zone organized as a crown of helper (CD4+) and cytotoxic T cells (CD8+) in interaction with some mature DC (DCLAMP+) and macrophages (CD68+). These non-GC TLS also contained HLADR+ cells allowing antigen presentation (**Figures 3A, B, D**).

GC-like TLS are defined as LA with mature FDCs (CD21+CD23+) and a functional and organized GC, corresponding to the PFL-TLS stage (8). This GC had proliferative (Ki67+) GC B cells (CD23+) expressing AID, involved in somatic hypermutations and class switch recombination, as well as Bcl6+ cells, Bcl6 being a transcription factor involved in GC B cell maturation and follicular T cell differentiation. In addition to FDCs (CD21+CD23+podoplanin+), the proliferative center was surrounded by mantle zone activated B cells (IgD+CD20+) and contained some macrophages (CD68+) as well as CD4+ and CD8+ T cells (CD3+). We also observed a crown of follicular helper T cells (Bcl6+CXCR5+CD3+) and plasma cells (CD38+CD138+) often surrounding GC indicative of an ongoing humoral and cytotoxic immune response, by the production of IgG and IgA (**Figures 3A, B, E**).

This classification better suits to the definition of TLS, i.e an organized aggregate of immune cells, where the first stage called LA, corresponding to the E-TLS stage in Silina’s work (8), is not already considered as a TLS, since nothing can predict the evolution of the disorganized LA into an organized structure meeting the definition of TLS.

**Figure 4:**
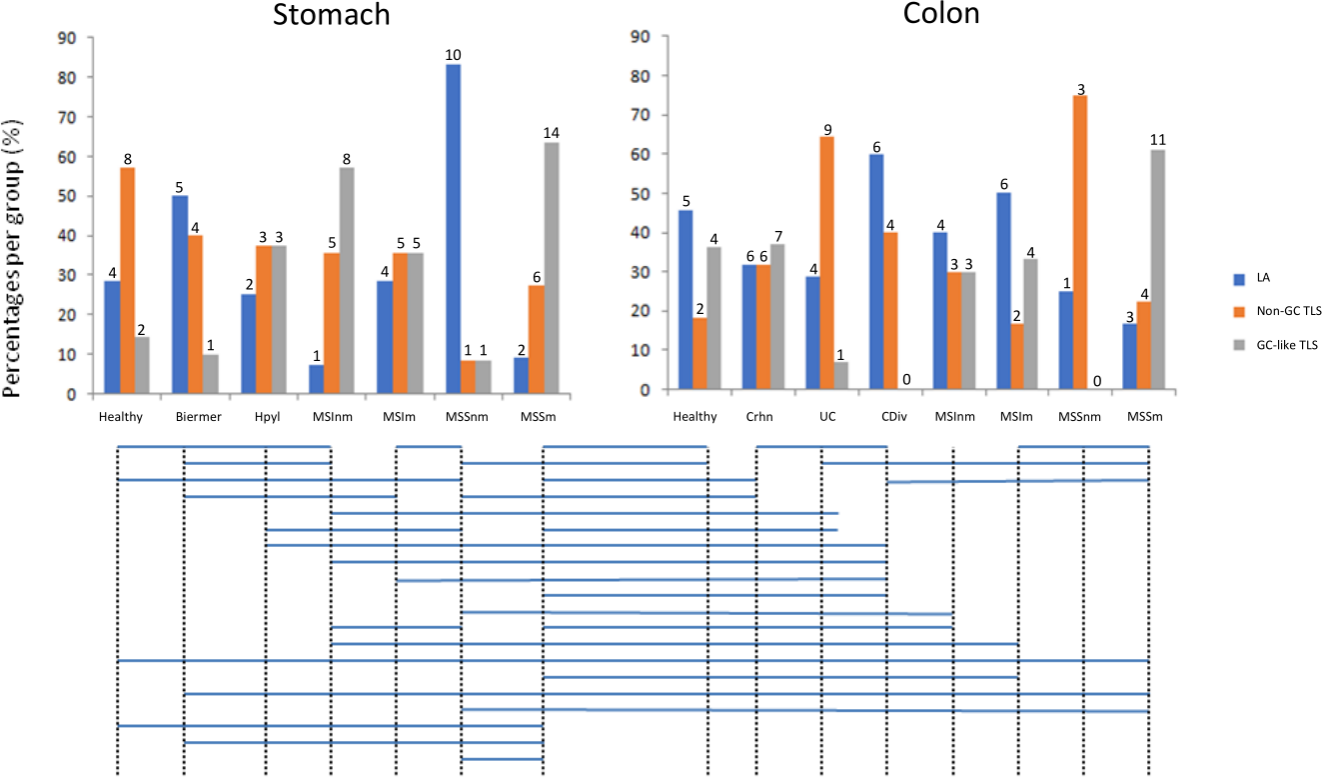
Distributions of the three stages of TLS across the different sample groups. Groups with significantly different distributions (p<0.05) are joined by horizontal bars under the histograms. The quantitative numbers of TLS are represented above each bar. LA: lymphoid aggregates; Non-GC TLS: non-germinal center TLS; GC-like TLS: germinal center-like TLS; groups A to E2 correspond to gastric samples: A: no gastric disease, B: Biermer’s autoimmune gastritis, C: Helicobacter pylori-related gastritis, D1: microsatellite instable gastric cancers without nodal metastasis, D2: microsatellite instable gastric cancers with nodal metastasis, E1: microsatellite stable gastric cancers without nodal metastasis, E2 microsatellite stable gastric cancers with nodal metastasis; groups 1 to 6B correspond to colonic samples: 1: Peyer’s patches, 2: Crohn’s disease, 3: ulcerative colitis, 4: chronic diverticulitis, 5A: microsatellite instable colonic cancers without nodal metastasis, 5B: microsatellite instable colonic cancers with nodal metastasis, 6A: microsatellite stable colonic cancers without nodal metastasis, 6B: microsatellite stable colonic cancers with nodal metastasis.

Among different TLS from the same patient, we observed the co-existence of the three stages of TLS evolution (**Figure 3A**), even with the 3-markers-based classification (**Figure 3B**). We also observed significantly different distributions of these three stages between different gastric and colonic pathological sample groups (**Figure 4**) (Kruskal-Wallis test, p-value=0.000115). E.g. gastric MSS adenocarcinomas with regional nodal metastatic spreading had more GC-like TLS whereas LA were predominant in gastric MSS adenocarcinomas without regional metastasis.

## 4 Discussion

In recent years, TLS have been studied and described in different tissues and organs, being the subject of a growing number of publications, underlining the evidence of the potential value of TLS as prognostic and predictive biomarkers to anticipate the aggressiveness of a cancer and responses to anti-cancer treatments including immune checkpoint inhibitors (5, 20–26). To date, TLS are not quantified nor qualified in the pathological examination and, despite their well-demonstrated relation to diseases evolution, they are not taken into account for any treatment decisions. Before TLS characterization can be assessed by pathologists for a treatment-contributive purpose, several points have to be addressed.

Our study is the first one to use IMC to concomitantly analyze 39 markers of cell populations and TLS related functions to answer questions related to the composition and organization of TLS in various digestive diseases, gastric and colonic ones, cancerous and non-cancerous ones. Through this study, unsupervised analyses of the 39 markers suggests that TLS compositionis not specific of a given disease; indeed, the more pronounced clustering of TLS phenotypes per patient supports that TLS compositions may reflect each patient’s proper immune reaction to a given pathological process.

Comparisons in terms of cell contents and organizations between SLO and TLS confirm that ectopic TLS remain less organized than SLO. Of note, based on our scoring method, Peyer’s patches (that are considered as SLO and not TLS) seem to be closer to TLS than LN in terms of organization; this could emphasize the role of the capsule in the superior organization of LN in comparison with non-encapsulated and less organized lymphoid structures like Peyer’s patches and TLS. Nevertheless, correlations between markers are consistent with a progressive organization through a maturation process from non-organized and non-functional TLS to highly organized and functional ones. This is concordant with the maturation of TLS reported in the literature towards several different sets of markers, most of them being included in our IMC TLS-dedicated panel. For future clinical applications, routine assessment of TLS maturation will presumably not be feasible through highly-multiplexed but also money- and time-consuming analyses like IMC. Thus, fewer markers and appropriate inter-grade boundaries are needed to permit the grading of TLS through routine pathological methods. As proposed by Silina and Posh, TLS in our series could be separated into our three categories on the basis of only three markers CD20, CD21 and CD23 for the characterization of immatures (CD21+) and matures FDCs (CD21+CD23+) and GC B cells (CD20+) (**Figure 3B**) (5–8). Towards this three-stages classification, we observed inter-organs and inter-diseases significant differences in the maturation degree of TLS. The small number of TLS and samples in various diseases analyzed through IMC in our study prevents us to draw formal conclusions and additional work with larger numbers of TLS and samples in series of patients with clinical data related to diseases evolution and responses to treatments will be necessary to investigate whether this maturation classification of TLS could consist in novel clinically relevant biomarkers. Nonetheless, this 3-markers approach, correlated with the evolution of other GC IMC markers in our study, appears as a valuable way to class TLS and would be easy to apply through routine pathology methods like multicolor-IHC. This shift towards IHC will also have the advantage to allow analyses of whole tissue slide images (WSI), hardly feasible for cost and technical reasons in IMC. These WSI analyses will provide additional data about TLS, notably on the location and count of TLS in cancer tissues.

## 5 Conclusion

This study using in-depth highly multiplexed IMC underlines the complexity and heterogeneity of TLs between organs and diseases at the level of cell compositions and interactions. Nevertheless, simpler and more implementable strategies based on a few markers to grade TLS in three maturation stages of organization and functional significance are rendered possible by the correlations between markers of our large panel. Thanks to this transfer from high-level to few markers’ classification, more studies will be feasible from now on in order to investigate the pathophysiological and medical relevance of grading, counting and locating TLS in pathological tissues, in digestive but also non-digestive organs as well as in cancer and non-cancer diseases.

## Data availability statement

The raw data supporting the conclusions of this article will be made available by the authors, without undue reservation.

## Ethics statement

Written informed consent was obtained from the individual(s) for the publication of any potentially identifiable images or data included in this article.

## Author contributions

Involved in the conception or design of the work: MR, J-OP, AU, DC. The conception of the panel: MR, DB-G, PH, AU. Data acquisition: MR, DB-G. Analysis of the data: MR, PH, CD, SG. All contributing authors critically revised the article, contributed to the article, and approved the submitted version.
