# Supplementary material for: Deciphering the maturation of tertiary lymphoid structures in cancer and inflammatory diseases of the digestive tract using imaging mass cytometry
Source: Front Immunol. 2023 Apr 18;14:1147480. doi: 10.3389/fimmu.2023.1147480 (PMC10151544; doi:10.3389/fimmu.2023.1147480)
Supplement: Supplementary file 1 [file DataSheet_1.pdf]

Supplementary Material

Deciphering the maturation of tertiary lymphoid structures in cancer and inflammatory diseases of the digestive tract using imaging mass cytometry

Marion Le Rochais*, Patrice Hémon, Danivanh Ben-guigui, Soizic Garaud, Christelle Le Dantec, Jacques-Olivier Pers, Divi Cornec, Arnaud Uguen

*** Correspondence:** Corresponding Author: email@uni.edu

1 Supplementary Figures

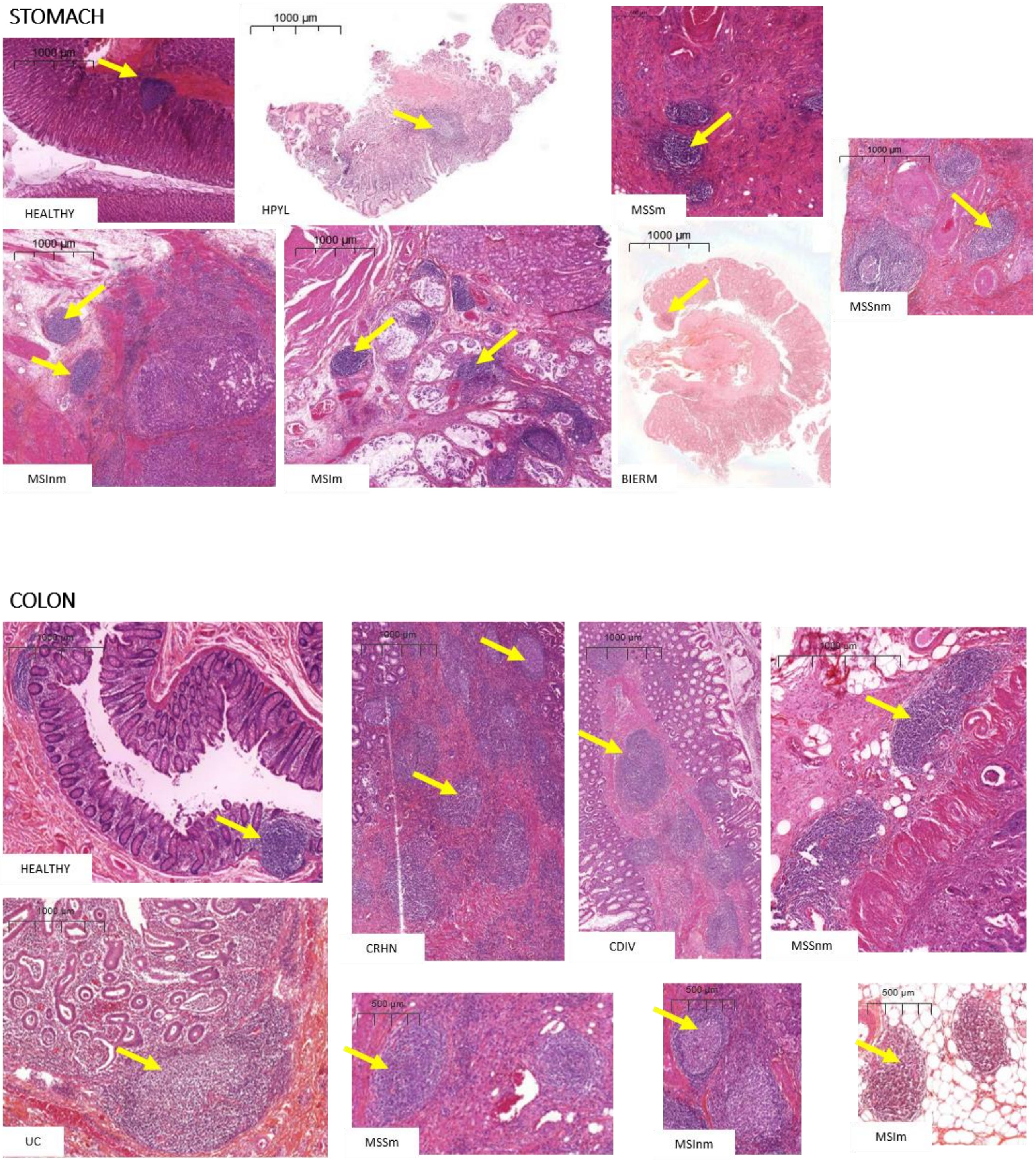

Supplementary Figure 1. HES scans of the different conditions in the stomach and in the colon, yellow arrows indicate TLS observed and chosen by the pathologist.

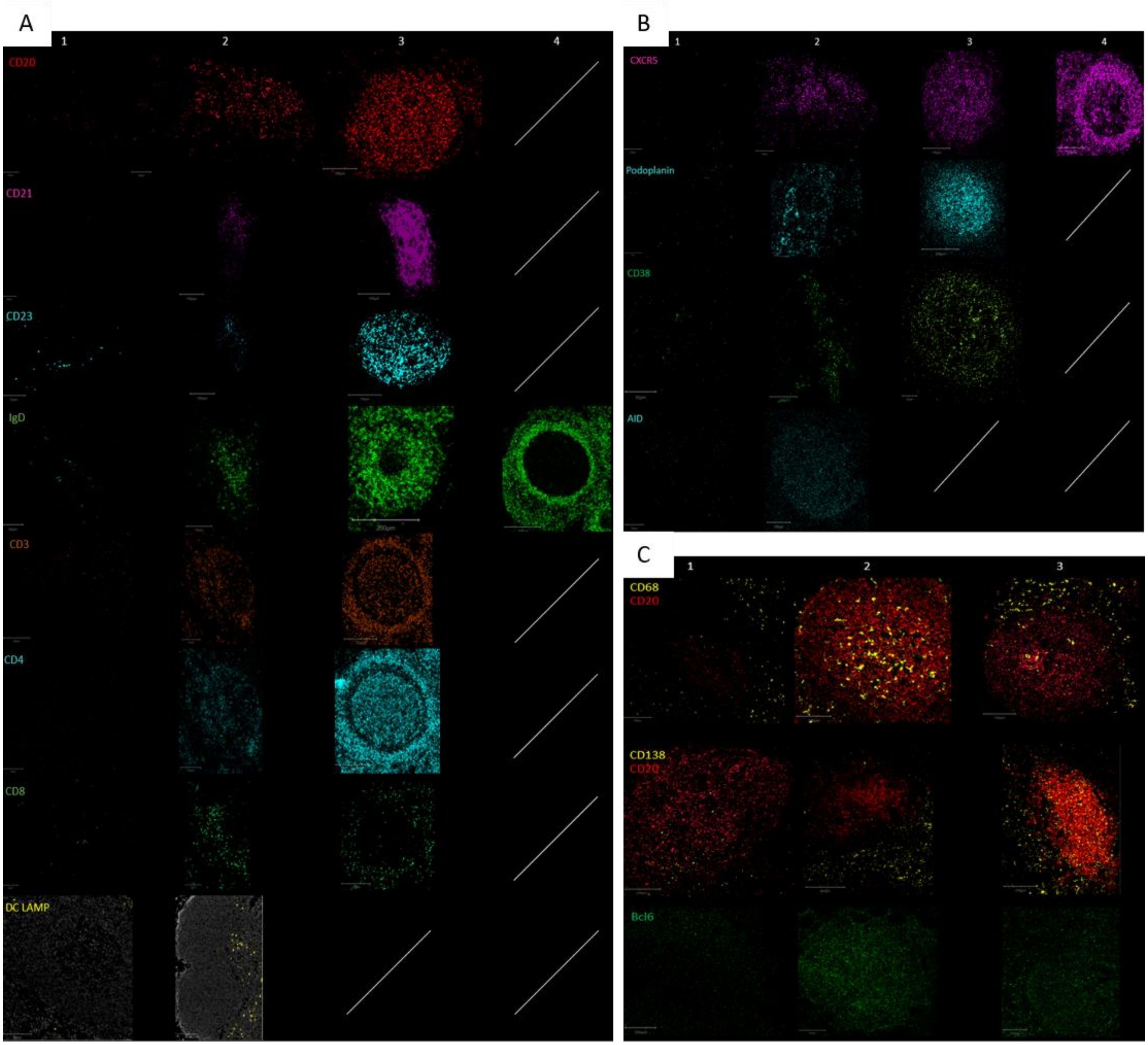

Supplementary Figure 2. Grading of the morphological assessment of the TLS according to the morphological organization of (A) the 8 structural markers of a germinal center (B) the 4 functional markers (C) the 3 markers for the nodular interactions. The white oblique lines indicate that this score does not exist for the marker considered.

A

No correlation **-0,03** → **1** Correlation

	CD20	CD21	CD23	IgD	CD3	CD4	CD8	DC LAMP	CXCR5	Podoplanin	CD68	CD38	CD138	AID	Bcl6
CD20	1	0,657	0,486	0,610	0,586	0,625	0,439	0,237	0,504	0,570	0,326	0,253	0,223	0,322	0,246
CD21	0,657	1	0,552	0,561	0,338	0,356	0,223	0,097	0,467	0,508	0,228	0,324	0,211	0,296	0,260
CD23	0,486	0,552	1	0,619	0,382	0,416	0,281	0,211	0,258	0,476	0,248	0,371	0,184	0,537	0,257
IgD	0,610	0,561	0,619	1	0,452	0,531	0,254	0,227	0,398	0,524	0,272	0,274	0,152	0,491	0,201
CD3	0,586	0,338	0,382	0,452	1	0,898	0,668	0,369	0,396	0,446	0,216	0,261	0,271	0,254	0,193
CD4	0,625	0,356	0,416	0,531	0,898	1	0,746	0,415	0,468	0,456	0,224	0,265	0,192	0,249	0,165
CD8	0,439	0,223	0,281	0,254	0,668	0,746	1	0,323	0,469	0,162	0,194	0,174	0,266	0,163	0,231
DC LAMP	0,237	0,097	0,211	0,227	0,369	0,415	0,323	1	0,147	0,212	0,008	0,307	0,232	0,258	0,104
CXCR5	0,504	0,467	0,258	0,398	0,396	0,468	0,469	0,147	1	0,332	0,331	-0,011	0,293	0,181	0,283
Podoplanin	0,570	0,508	0,476	0,524	0,446	0,456	0,162	0,212	0,332	1	0,340	0,279	0,180	0,275	0,371
CD68	0,326	0,228	0,248	0,272	0,216	0,224	0,194	0,008	0,331	0,340	1	-0,033	0,016	0,178	0,410
CD38	0,253	0,324	0,371	0,274	0,261	0,265	0,174	0,307	-0,011	0,279	-0,033	1	0,291	0,243	0,062
CD138	0,223	0,211	0,184	0,152	0,271	0,192	0,266	0,232	0,293	0,180	0,016	0,291	1	0,239	0,282
AID	0,322	0,296	0,537	0,491	0,254	0,249	0,163	0,258	0,181	0,275	0,178	0,243	0,239	1	0,241
Bcl6	0,246	0,260	0,257	0,201	0,193	0,165	0,231	0,104	0,283	0,371	0,410	0,062	0,282	0,241	1

Low + correlation (>0,5)

CD21/ CD20	CD21/ CD23	CD20/ IgD	CD20/ CD3	CD20/ CD4	CD20/ CXCR5	CD20/ Podo	CD21/ IgD	CD21/ podo	CD23/ AID	CD23/ IgD	IgD/ CD4	IgD/ podo	CD3/ CD8
0,657	0,552	0,610	0,586	0,625	0,504	0,570	0,561	0,570	0,537	0,619	0,531	0,524	0,668

High + correlation (>0,7)

CD3/CD4	CD4/CD8
0,898	0,746

B

No correlation **-0,01** → **1** Correlation

	CD20	CD21	CD23	IgD	CD3	CD4	CD8	DC LAMP	CXCR5	Podoplanin	CD38	AID	Bcl6	CD138	CD68
CD20	1	0,515	0,528	0,616	0,539	0,483	0,543	0,305	0,496	0,450	0,376	0,457	0,386	0,049	0,433
CD21	0,515	1	0,725	0,567	0,328	0,262	0,378	0,125	0,419	0,460	0,221	0,400	0,144	0,042	0,382
CD23	0,528	0,725	1	0,585	0,491	0,372	0,544	0,235	0,561	0,399	0,330	0,474	0,148	0,074	0,306
IgD	0,616	0,567	0,585	1	0,303	0,192	0,335	0,111	0,496	0,365	0,236	0,569	0,086	0,077	0,403
CD3	0,539	0,328	0,491	0,303	1	0,766	0,804	0,522	0,437	0,346	0,482	0,356	0,376	-0,014	0,338
CD4	0,483	0,262	0,372	0,192	0,766	1	0,806	0,460	0,390	0,343	0,365	0,349	0,426	-0,008	0,336
CD8	0,543	0,378	0,544	0,335	0,804	0,806	1	0,487	0,509	0,405	0,468	0,371	0,424	-0,006	0,422
DC LAMP	0,305	0,125	0,235	0,111	0,522	0,460	0,487	1	0,323	0,149	0,385	0,100	0,347	-0,097	0,216
CXCR5	0,496	0,419	0,561	0,496	0,437	0,390	0,509	0,323	1	0,447	0,405	0,582	0,297	0,058	0,415
Podoplanin	0,450	0,460	0,399	0,365	0,346	0,343	0,405	0,149	0,447	1	0,330	0,310	0,280	0,197	0,441
CD38	0,376	0,221	0,330	0,236	0,482	0,365	0,468	0,385	0,405	0,330	1	0,233	0,359	0,199	0,276
AID	0,457	0,400	0,474	0,569	0,356	0,349	0,371	0,100	0,582	0,310	0,233	1	0,171	-0,001	0,386
Bcl6	0,386	0,144	0,148	0,086	0,376	0,426	0,424	0,347	0,297	0,280	0,359	0,171	1	0,147	0,329
CD138	0,049	0,042	0,074	0,077	-0,014	-0,008	-0,006	-0,097	0,058	0,197	0,199	-0,001	0,147	1	0,171
CD68	0,433	0,382	0,306	0,403	0,338	0,336	0,422	0,216	0,415	0,441	0,276	0,386	0,329	0,171	1

Low + correlation (>0,5)

CD21/ CD20	CD20/ CD23	CD20/ IgD	CD20/ CD3	CD20/ CD4	CD21/ IgD	CD23/ IgD	CD23/ CD8	CD23/ CXCR5	IgD/ AID	CD3/ DCLAMP	CD8/ CXCR5	CXCR5/ AID
0,515	0,528	0,616	0,539	0,543	0,567	0,585	0,544	0,561	0,569	0,522	0,509	0,582

High + correlation (>0,7)

CD21/CD23	CD3/CD4	CD3/CD8	CD4/CD8
0,725	0,766	0,804	0,806

Supplementary Figure 3. Matrix of the correlation scores of the 15 markers characterizing GC. Positive and negative correlations are shown in green and red, respectively **A)** In the stomach **B)** In the colon.
